# Elevated Hypothalamic Glucocorticoid Levels Are Associated With Obesity and Hyperphagia in Male Mice

**DOI:** 10.1210/en.2016-1571

**Published:** 2016-09-20

**Authors:** Charlotte Sefton, Erika Harno, Alison Davies, Helen Small, Tiffany-Jayne Allen, Jonathan R. Wray, Catherine B. Lawrence, Anthony P. Coll, Anne White

**Affiliations:** Faculty of Biology (C.S., E.H., A.D., T.-J.A., J.R.W., C.B.L., A.W.), Medicine and Health, University of Manchester, Manchester M13 9PT, United Kingdom; Cancer Research UK Manchester Institute (H.S.), University of Manchester, Manchester M20 4BX, United Kingdom; and University of Cambridge Metabolic Research Laboratories and MRC Metabolic Diseases Unit (A.P.C.), Wellcome-MRC Institute of Metabolic Science, Addenbrooke's Hospital, Cambridge CB2 0QQ, United Kingdom

## Abstract

Glucocorticoid (Gc) excess, from endogenous overproduction in disorders of the hypothalamic-pituitary-adrenal axis or exogenous medical therapy, is recognized to cause adverse metabolic side effects. The Gc receptor (GR) is widely expressed throughout the body, including brain regions such as the hypothalamus. However, the extent to which chronic Gcs affect Gc concentrations in the hypothalamus and impact on GR and target genes is unknown. To investigate this, we used a murine model of corticosterone (Cort)-induced obesity and analyzed Cort levels in the hypothalamus and expression of genes relevant to Gc action. Mice were administered Cort (75 μg/mL) or ethanol (1%, vehicle) in drinking water for 4 weeks. Cort-treated mice had increased body weight, food intake, and adiposity. As expected, Cort increased plasma Cort levels at both zeitgeber time 1 and zeitgeber time 13, ablating the diurnal rhythm. Liquid chromatography dual tandem mass spectrometry revealed a 4-fold increase in hypothalamic Cort, which correlated with circulating levels and concentrations of Cort in other brain regions. This occurred despite decreased 11β-hydroxysteroid dehydrogenase (*Hsd11b1*) expression, the gene encoding the enzyme that regenerates active Gcs, whereas efflux transporter *Abcb1* mRNA was unaltered. In addition, although Cort decreased hypothalamic GR (*Nr3c1*) expression 2-fold, the Gc-induced leucine zipper (*Tsc22d3*) mRNA increased, which indicated elevated GR activation. In keeping with the development of hyperphagia and obesity, Cort increased *Agrp*, but there were no changes in *Pomc*, *Npy*, or *Cart* mRNA in the hypothalamus. In summary, chronic Cort treatment causes chronic increases in hypothalamic Cort levels and a persistent elevation in *Agrp*, a mediator in the development of metabolic disturbances.

Excess glucocorticoids (Gcs) produced by uncontrolled overactivity of the hypothalamic-pituitary-adrenal (HPA) axis, such as in Cushing's syndrome, lead to the development of a number of metabolic disorders. However, on a population scale, a much larger burden of Gc-induced metabolic problems occurs as a result of long-term exogenous Gc use for a wide range of inflammatory and malignant conditions. Although some of these adverse metabolic consequences can be ameliorated, for example, bisphosphonate treatment for steroid-induced osteoporosis, the Gc-induced disorders of increased body weight and abnormal glucose metabolism are often difficult to treat effectively. There is emerging evidence that 1 in every 150–200 patients Gc treated for rheumatoid arthritis develops diabetes, with a dose-related increase of 25%–30% in the prevalence of diabetes (1). An understanding of the site of action and mechanisms driving these side effects is therefore essential in considering how to ameliorate the unwanted actions of this successful class of therapeutic agents.

The peripheral mechanisms involved in the development of Gc-induced metabolic disorders have been widely documented. Gc actions in liver, adipose tissue, and skeletal muscle highlight their regulatory role in carbohydrate, lipid, and protein metabolism (reviewed in Ref. 2). In addition, the tissue-specific regeneration of Gcs within liver and adipose tissue provides an important mechanism for their actions within the periphery (3). Gc receptors (GRs) are also widely distributed throughout the brain, and yet the contribution of Gc actions in brain regions known to regulate energy balance is often ignored when considering the effects of excess Gcs on metabolic regulation.

Pharmacological concentrations of Gcs acting in the hypothalamus are ideal candidates for generating abnormal networking, which could lead to metabolic side effects. Gcs are known to regulate a range of orexigenic and anorexigenic neuropeptides in centers of the brain with a role in the control of food intake and body weight, although present data are somewhat contradictory. Dependent on the method and duration of corticosterone (Cort) administration, proopiomelanocortin (*Pomc*) expression has both increased (4, 5) and decreased (6) after treatment. Similarly agouti-related protein (*Agrp*) expression follows this inconsistency in response to Cort treatment (7, 8). Although the Gc-response elements (GREs) are present within the promotor regions of *Agrp* (9), *Pomc* (10), and neuropeptide Y (*Npy*) (11), the effect of chronic Gc treatment is still unclear.

It is dogma that Gcs act on corticotrophin-releasing hormone neurons as part of the self-regulatory feedback of the HPA axis, consequently down-regulating GR expression in the paraventricular nucleus (PVN). To prevent accumulation of Gcs in the brain the efflux transporter (multi-drug resistance p-glycoprotein) removes Gcs from the brain (12), whilst conversely the enzyme 11β-hydroxysteroid dehydrogenase type 1 (11β-HSD1) converts inactive 11-dehydrocorticosterone (11-DHC) to Cort, contributing to the levels of Cort in the hypothalamus. However, the effects of chronic Cort treatment on these mechanisms, which control the absolute concentrations of Cort in the hypothalamus, are unknown.

To investigate the effects of chronic Gc treatment on hypothalamic regulation of energy balance requires a well-characterized model. Unfortunately, there is a history of conflicting data describing the effects of Cort on body weight in rodent models. The effects of exogenous Cort treatment can increase body weight (13, 14) or decrease it (15, 16).

An elegant study in 2010 used a translational model of Cort administration in the drinking water to mimic the excess Gcs seen in patients on long-term Gc treatment (17). However, the levels of Gcs that are able to translocate to and remain in the brain to regulate central metabolic effects are unclear. Therefore, this study aimed to analyze Gc levels within the hypothalamus after Cort treatment at doses that cause metabolic side effects. We also evaluated the effects of Gcs on genes regulating hypothalamic Gc concentrations and on Gc target genes including those known to contribute to an adverse metabolic phenotype.

## Materials and Methods

### Animal husbandry and administration of Cort treatment

Ten-week-old male C57Bl/6J mice (Charles River) were singly housed under a constant 12-hour light, 12-hour dark cycle (lights on 7 am, zeitgeber time [ZT]0, lights off 7 pm, ZT12), with an ambient temperature of 23 ± 1°C and a humidity of approximately 40%. Food and water were available ad libitum through all experiments. All experiments were performed in accordance with United Kingdom Animals (Scientific Procedures Act, 1986) using procedures approved by The University of Manchester Ethical Review Panel.

Food intake and body weight measurements were measured twice weekly throughout the experiment. After 3 weeks of baseline, mice were randomly assigned to a treatment group by body weight and administered either Cort (75 μg/mL; Sigma-Aldrich) dissolved in 1% ethanol or vehicle (1% ethanol) for 24 hours, 48 hours, or 4 weeks. Separate cohorts were used to allow the analysis of both hypothalamic Cort levels (liquid chromatography dual tandem mass spectrometry [LC-MS/MS], n = 12 per group) and gene expression analysis (quantitative reverse transcription PCR [qRT-PCR], n = 8 per group, in situ n = 3 per group). At the end of each study, blood was taken by tail-prick sampling for analysis of circulating Cort: plasma was removed and stored at −80° for future analysis. Immediately after sampling, mice were culled by cervical dislocation. Tissues were dissected and snap-frozen on dry ice for future analysis. For mRNA analysis, the whole hypothalamus (∼20 mg) was removed immediately from the ventral side of the brain. Microdissection scissors cut immediately caudal to the optic chiasm. The dissection was limited laterally by the hypothalamic sulci and dorsally by the mammilothalamic tract. The entire hypothalamus was removed, including the arcuate nucleus, ventromedial nucleus, dorsomedial nucleus, and PVN, and stored in RNAlater for future analysis.

### Plasma Cort quantification

Plasma Cort levels were quantified using an ELISA according to the manufacturer's instructions (for 4-wk Cort treatment, Cayman, Cambridge Bioscience; for 24- and 48-h Cort treatment, Abcam, because the Cayman kit was discontinued).

### Tissue Cort and 11-DHC quantification

Tissue Cort levels were measured in hypothalamic-enriched and extrahypothalamic regions (rest of the brain) by LC-MS/MS. A hypothalamic-enriched region was dissected from the ventral side of the frozen brain (∼50 mg). Two coronal microdissection cuts were made at the level of the optic chiasm and the mammillary bodies, the brain was then rotated, and 2 more cuts were made to dissect out the hypothalamus as a rectangle. The rest of the brain was homogenized as one and is described as the extrahypothalamic region (∼375 mg). Both hypothalamic-enriched and extrahypothalamic regions were homogenized in sterile water (200 mg/mL) using a rotor homogenizer. A 100-μL sample aliquot was enriched with Cort-D8 major as internal standard to a concentration of 10 μg/mL. A liquid/liquid extraction was performed followed by a solid phase extraction. Briefly, 500 μL of ethyl acetate were added to each sample, aspirated, and then centrifuged (500 rpm, 10 min) to separate the layers. The aqueous layer was discarded while the solvent layer was blown to dryness under nitrogen and then reconstituted in 30% methanol. A solid phase extraction was performed using a C-18 plate (Strata C18-E; Phenomenex), which was conditioned with methanol and water. Samples were loaded in 30% methanol, washed with double distilled H_2_O and 30% methanol before elution in 100% methanol (2× 250 μL). The eluents were dried under nitrogen and reconstituted in 50% mobile phase A/50% mobile phase B. Mobile phase A was 2mM ammonium acetate in methanol/water (10/90 vol/vol) containing 0.027% formic acid. Mobile phase B was 2mM ammonium acetate in methanol/water (90/10 vol/vol) containing 0.027% formic acid.

Chromatographic separation of Cort and 11-DHC was performed using reverse phase chromatography (Kinetex 5-μm XB-C18 100 Å, 50 × 2.1 mm; Phenomenex) on an Agilent 1200 binary pump HPLC system running a gradient from 100% A to 100% B over 1.5 minutes, hold for 1.5 minutes, then immediately back to 100% A and hold for 3.5 minutes. Total run time was 6.5 minutes with flow rate of 500 μL/min and an injection volume of 10 μL. The system was run at room temperature.

The chromatography system was coupled to a Sciex API4000 Qtrap mass spectrometer. The instrument was operated in multiple reaction monitoring. Cort was monitored in positive ion mode; the following parameters were implemented in the ion source: spray voltage, 4.5 kV, temperature, 550°C, curtain gas at 20, gas 1 at 50, and gas 2 at 60. The transitions used were Cort: 347.1/329.3 with collison energy (CE) = 22, orifice (DP) = 37, and cell exit potential (CXP) = 15; D8-Cort (internal standard): 355.3/73 with CE = 50, DP = 117, and CXP = 15.

11-DHC was monitored in negative ion mode; the following parameters were implemented in the ion source: spray voltage, −4.5 kV, temperature, 550°C, curtain gas at 20, gas 1 at 50, and gas 2 at 60. The transitions used were 11-DHC: 343.1/328.0 with CE = −28, DP = −100, and CXP = −15; D8-Cort (internal standard): 353.1/246.1 with CE = −25, DP = −100, and CXP = −15.

Data were processed using Analyst Software (Applied Biosystems) and signal intensities obtained by standard peak integration methods. Quantitations were performed by comparison against a standard curve generated from multiple dilutions of brain tissue spiked with analyte of interest in methanol (10–0.01 ng/μL). Final analyte quantification was derived from a mean of 2 biological replicates. The lower limit of accurate quantification was set at a level twice the peak area measured in blank brain tissue, to ensure robust and confident discrimination of analytes from low-level background peaks which may coelute. The mean lower limit of accurate quantification was 0.3-ng/mL Cort, 0.5-ng/mL 11-DHC. When samples were measured as below limit of quantification statistical analysis was performed using a value of 0.

### Quantification of mRNA by real-time quantitative PCR

RNA was extracted using an RNeasy mini kit (QIAGEN) with on column genomic DNA digestion according to the manufacturer's protocol. RNA integrity and quantification was confirmed using a NanoDrop ND2000 (Thermo Scientific). Transcript levels were determined by reverse transcription and qRT-PCR on a Prism 7900HT Sequence Detection System (Applied Biosystems) with either TaqMan Gene Expression Assays and TaqMan RNA-to-C_T_ 1 step kit (Applied Biosystems) or SYBR assays designed with primer-BLAST software (NCBI) and Power SYBR Green RNA-to-C_T_ 1 step kit (Applied Biosystems). Relative quantification was achieved using a standard curve approach with normalization to the reference genes, *Hprt* or *Tbp* for TaqMan or SYBR assays, respectively, and nomination of the vehicle group as calibrator. The primer sequences used in this study were: *Abcb1* forward 5′-TTTGGCAAAGCCGGAGAGAT-3′ and reverse 5′-CCAGCTTATATCCTGTCTCAGCA3′; *Agrp* Mm00475829_g1; *Cartpt* Mm04210469_m1; FK506-binding protein 5 (*Fkbp5*) forward 5′-AGCAACGGTAAAAGTCCACCT-3′ and reverse 5′-TTCCCCAACAACGAACACCA-3′; *Hprt* Mm03024075_m1; *HSD11b1* Mm00476182_m1; *Npy* forward 5′-ATGCTAGGTAACAAGCGAATGG-3′ and reverse 5′-TGTCGCAGAGCGGAGTAGTAT-3′; *Nr3c1* forward 5′-AGCTCCCCCTGGTAGAGAC-3′ and reverse 5′-GGTGAAGACGCAGAAACCTTG-3′; *Pomc* forward 5′-ATGCCGAGATTCTGCTACAGT-3′ and reverse 5′-TCCAGCGAGAGGTCGAGTTT-3′; *Tbp* forward 5′-GGGAGAATCATGGACCAGAA-3′ and reverse 5′-GATGGGAATTCCAGGAGTCA-3′; and *Tsc22d3* forward 5′-GCAGGCCATGGACCTCGT GAAG-3′ and reverse 5′-TCAGGAGGGTGTTCTCGCGCT-3′.

### In situ hybridization

Frozen whole brains were sectioned (12 μm) using a cryostat freezing microtome. Coronal sections were taken of the entire hypothalamus from each animal starting from immediately before the PVN to the end of the third ventricle. Representative sections through the hypothalamus from each animal were then analyzed to ensure inclusion of all anatomical levels.

In situ riboprobes were generated by synthesis and subcloning of the sequence (GeneArt) into pGM-5ZF(+) vector (Promega). The resultant plasmid constructs were linearized using appropriate restriction enzymes, to generate templates for sense and antisense transcripts. Antisense and sense riboprobes were synthesized with an SP6/T7 in vitro transcription system (Promega) in the presence of ^33^P-uridine triphosphate (PerkinElmer). Probes were hybridized overnight at 60°C, and hybridization was visualized by film autoradiography (Kodak Bio-Max MR film; Kodak). Films were scanned using a CoolSNAP-Pro camera (Photometrics) while on a light box. Signal intensity was quantified by densitometry analysis of autoradiographs. Optical density (relative units) within the target region was calculated on each section with a minimum of 7 sections per animal, 3 animals per group.

### Statistical analysis

All data are represented as mean ± SEM and results considered statistically significant at *P* < .05. Hypothalamic, extrahypothalamic Cort levels and absolute tissue weights (unpaired *t* test), correlation analyses (Pearson's and Spearman's rank correlation), qRT-PCR (Mann-Whitney unpaired *t* test), circulating Cort, food intake, and body weight (two-way ANOVA). All statistical analyses were performed with GraphPad Prism version 6.00.

## Results

### Exogenous Cort treatment alters circulating Cort

**Figure 1.**
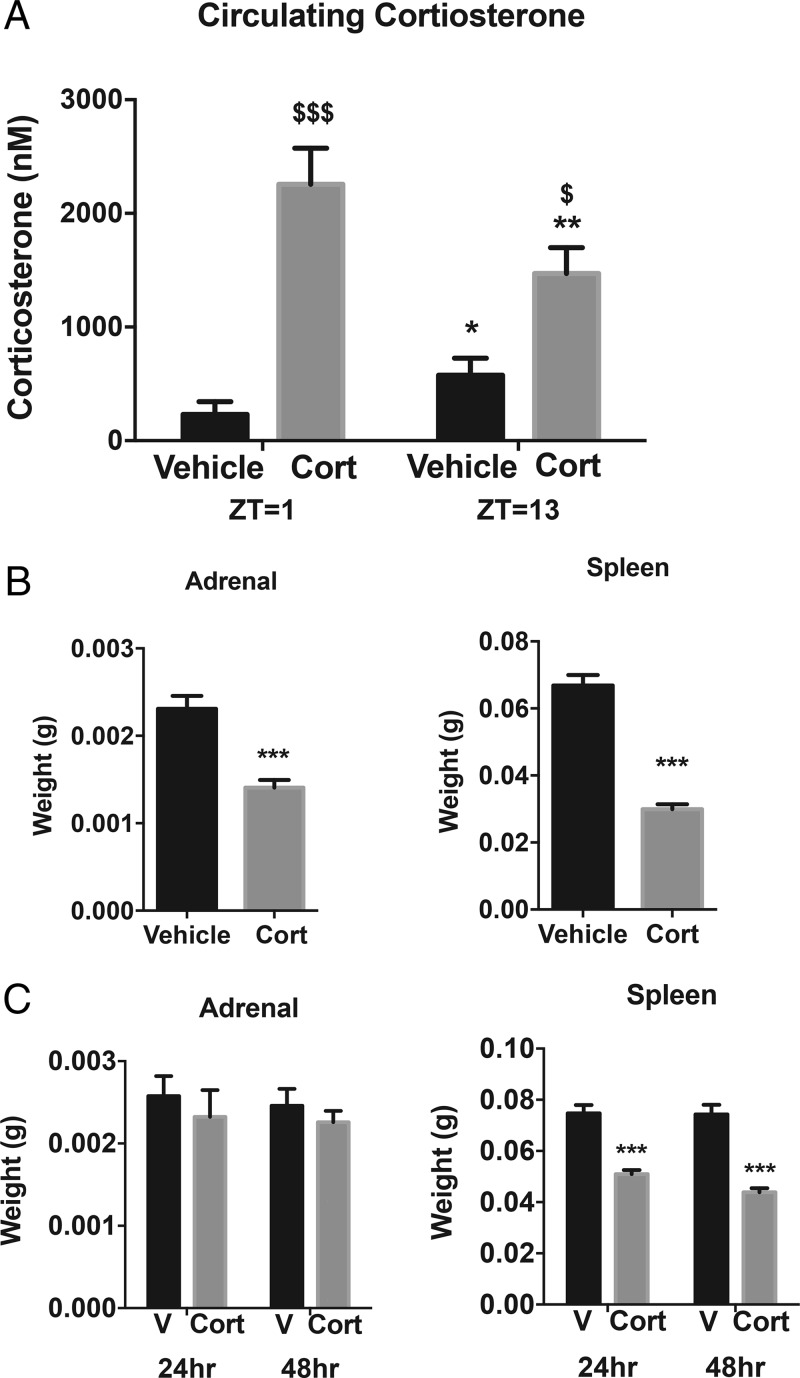
Exogenous Cort treatment alters circulating Cort. A, Plasma Cort levels (nM) quantified at zeitgeber time (ZT1), 7 am and ZT13, 7 pm after 4 weeks of Cort treatment ($, Cort vs vehicle [V]; *, ZT13 vs ZT1; n = 5–6/treatment group). B, Adrenal and spleen mass after 4 weeks of treatment, ZT4 (unpaired *t* test; ***, *P* < .0001, n = 11–12/treatment group). C, Adrenal and spleen mass after 24 and 48 hours of treatment, ZT4 (unpaired *t* test; ***, *P* < .0001, n = 12/treatment group).

After 4 weeks of exogenous Cort treatment, the plasma Cort was 9-fold higher in the Cort-treated mice at ZT1, and 2-fold higher at ZT13 compared with vehicle (*P* < .01) (Figure 1A). A reduction in adrenal weight was observed after 4 weeks of Cort treatment (*P* < .001) (Figure 1B), suggesting that the chronic increase in Gcs had suppressed the HPA axis. As expected, 4-week Cort treatment reduced spleen weight (*P* < .001) (Figure 1B), with this effect seen as early as 24 hours (*P* < .001) (Figure 1C). Whole pituitary *Nr3c1* (GR) mRNA expression was not altered after 4 weeks of Cort treatment (data not shown).

### Exogenous Cort treatment increases hypothalamic Cort levels after 24 and 48 hours

**Figure 2.**
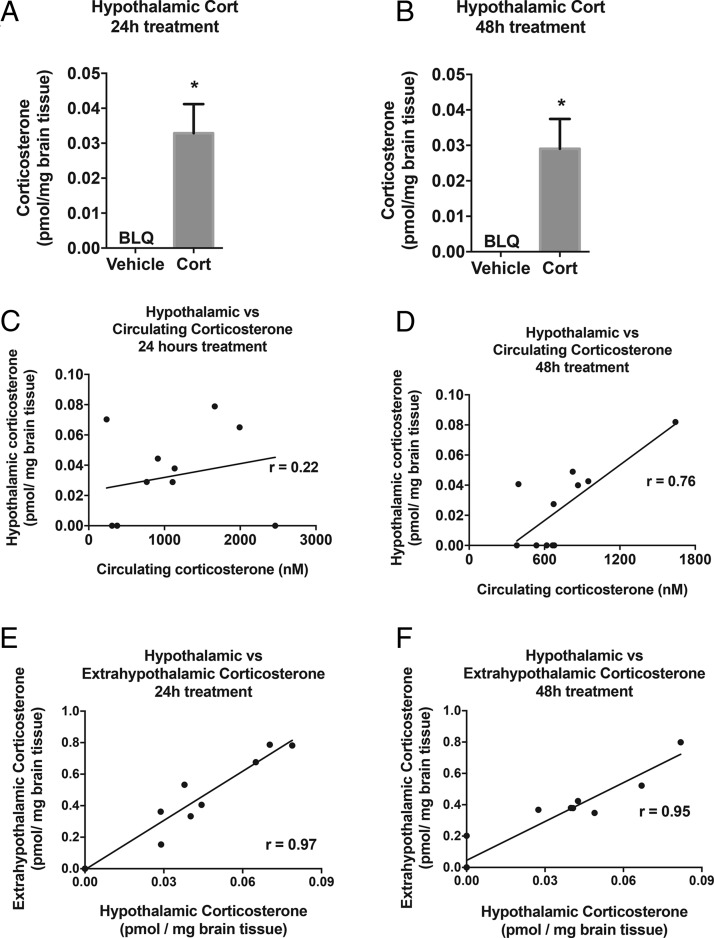
Exogenous Cort treatment increases hypothalamic Cort levels after 24 hours of treatment. LC-MS/MS quantification of hypothalamic Cort levels after (A) 24 hours of Cort treatment and (B) 48 hours of Cort treatment (unpaired *t* test; *, *P* < .05). Hypothalamic vs circulating Cort correlation analysis of Cort treatment group only after (C) 24 hours of Cort treatment (Pearson's correlation, *P* = .5, n = 12/treatment group) and (D) 48 hours of Cort treatment (Pearson's correlation; **, *P* < .01, n = 12/treatment group). Hypothalamic vs extrahypothalamic correlation analysis after (E) 24 hours and (F) 48 hours of Cort treatment (Pearson's correlation; ***, *P* < .001, n = 12/treatment group). BLQ, below limit of quantification.

To determine whether Cort treatment resulted in elevation of Cort in the hypothalamus within 24 hours or whether it gradually accumulated over time, levels were quantified after 24 and 48 hours of Cort treatment. Cort levels in the hypothalamus were increased compared with vehicle treated animals and this was similar after 24 and 48 hours of Cort treatment (*P* < .05) (Figure 2, A and B). Cort levels were correlated between hypothalamic and circulating Cort levels in the Cort-treated group (Figure 2, C [*P* = .5] and D [*P* < .01]). Similarly, within the rest of the brain (described as the extrahypothalamic region), Cort levels increased alongside hypothalamic Cort levels after 24 and 48 hours of Cort treatment (*P* < .001) (Figure 2, E and F). Vehicle-treated mice were not included in correlation analysis. 11-DHC can be converted to Cort by 11β-HSD1 in the hypothalamus, and therefore, 11-DHC levels were also assessed. However, hypothalamic and extrahypothalamic levels of 11-DHC were below the limit of quantification (data not shown).

### Four weeks of exogenous Cort treatment increases hypothalamic Cort levels

**Figure 3.**
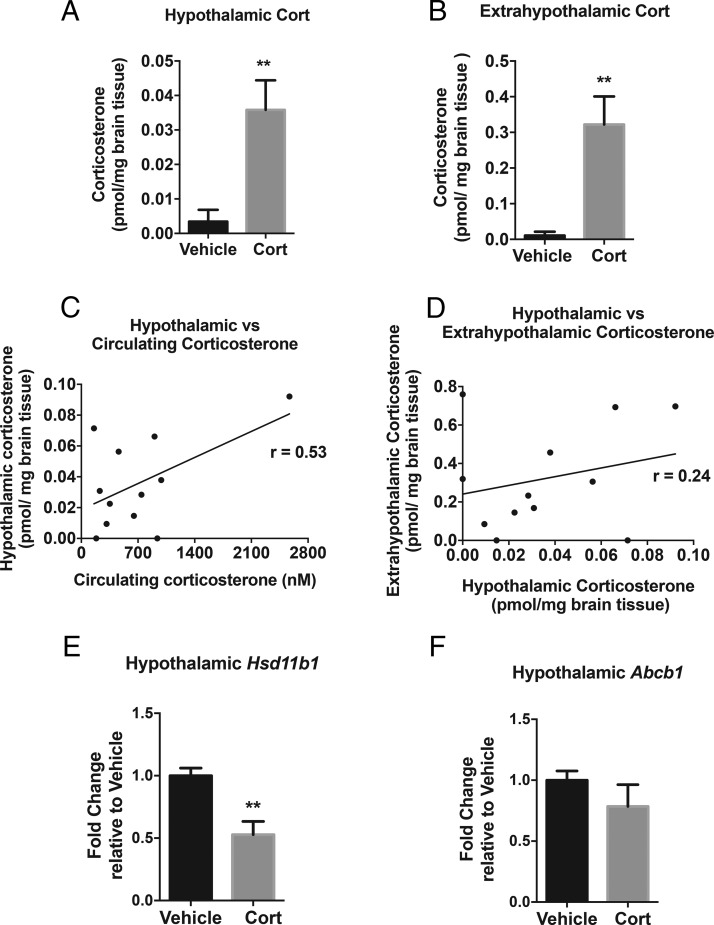
Exogenous Cort treatment increases hypothalamic Cort levels after 4 weeks. LC-MS/MS quantification of (A) hypothalamic and (B) extrahypothalamic Cort levels after 4 weeks of Cort treatment (unpaired *t* test; **, *P* < .01, n = 12/treatment group). C, Hypothalamic vs circulating Cort levels correlation analysis of Cort treatment group at time of death, ZT4 (Pearson's rank correlation; *P* = .07, n = 12/treatment group). D, Cort treatment correlation analysis of hypothalamic vs extrahypothalamic Cort levels (Pearson's rank correlation; *P* = .43, n = 12/treatment group). E, Hypothalamic *Hsd11b1* (11β-HSD1) and F, *Abcb1* (MDR1a) mRNA expression (Mann-Whitney *t* test; **, *P* < .01 n = 6–8/treatment group).

Hypothalamic Cort levels were increased 4-fold with 4 weeks of Cort treatment (*P* < .01) (Figure 3A). This increase is comparable with the increase seen after 24 and 48 hours. Similarly, extrahypothalamic Cort levels increased in mice treated with Cort (*P* < .01) (Figure 3B). Both hypothalamic (Figure 3C) and extrahypothalamic Cort levels (data not shown) were positively correlated with circulating Cort (*P* = .07) (Figure 3C) and with each other (*P* = .43) (Figure 3D). Only Cort-treated mice were included in correlation analysis. Hypothalamic and extrahypothalamic levels of 11-DHC were below the limit of quantification (data not shown).

There was a marked decrease in *Hsd11b1* (11β-HSD1) mRNA expression after Cort treatment, indicating reduced endogenous Cort regeneration from the inactive 11-DHC (*P* < .01) (Figure 3E). In comparison, *Abcb1* (gene for MDR1a, an efflux transporter known to remove Gcs across the blood brain barrier) did not change between treatment groups (Figure 3F).

### Four weeks of Cort increases GR activity in the hypothalamus

**Figure 4.**
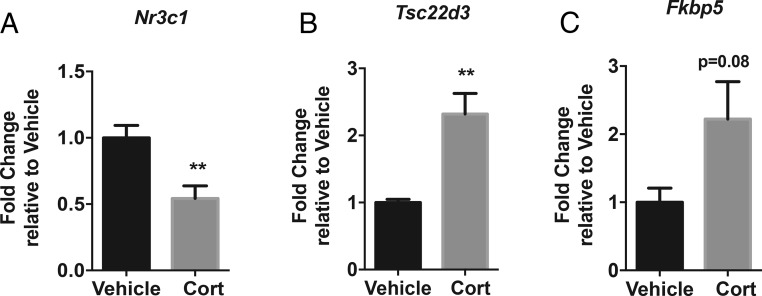
Chronic Cort increases GR activity in the hypothalamus. mRNA expression analysis of *Nr3c1* (GR) (A), *Tsc22d3* (Gilz) (B), and *Fkbp5* (C) in the whole hypothalamus after 4 weeks of Cort treatment (Mann-Whitney *t* test; **, *P* < .01, n = 5–8/treatment group).

After 4 weeks of Cort, the increased hypothalamic Cort levels decreased the hypothalamic mRNA expression of *Nr3c1* (GR) (*P* < .01) (Figure 4A). However, there was increased expression of *Tsc22d3* (Gc-induced leucine zipper [GILZ]), a transcription factor known to be regulated by Gcs (*P* < .01) (Figure 4B) and a trend towards an increase in *Fkbp5* within the hypothalamus (Figure 4C), indicating increased Gc activity. Mineralocorticoid receptor (*Nr3c2*) mRNA expression did not change with Cort treatment (data not shown). Hypothalamic *Crh* expression was below the limit of detection (data not shown) and therefore this data set has not been included.

### Exogenous Cort alters hypothalamic *AgRP* mRNA expression

**Figure 5.**
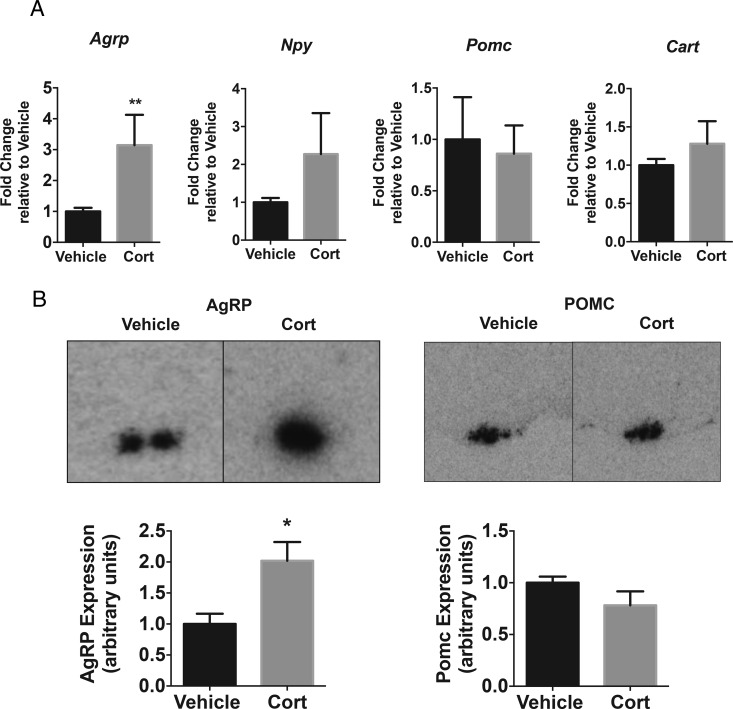
Exogenous Cort alters *Agrp* mRNA expression. A, *Agrp*, *Npy*, *Cart*, and *Pomc* mRNA expression analysis after 4 weeks of Cort treatment in the whole hypothalamus (Mann-Whitney *t* test; **, *P* < .01, n = 5–8/treatment group). B, Representative images and densitometry of in situ hybridization *AgRP* and *Pomc* expression in the arcuate nucleus (unpaired *t* test; **, *P* < .01, n = 3/treatment group).

Exogenous Cort treatment significantly increased *Agrp* mRNA expression in the hypothalamus (*P* < .01) (Figure 5A). However, hypothalamic mRNA expression of energy regulatory neuropeptides; *Npy*, *Pomc*, and cocaine and amphetamine-regulated transcript (*Cart*), were not altered after 4 weeks of treatment with Cort (Figure 5A). In situ hybridization confirmed increased *Agrp* expression throughout the arcuate nucleus, whereas *Pomc* expression was not altered after Cort treatment (*P* < .05 for *Agrp*) (Figure 5B).

**Figure 6.**
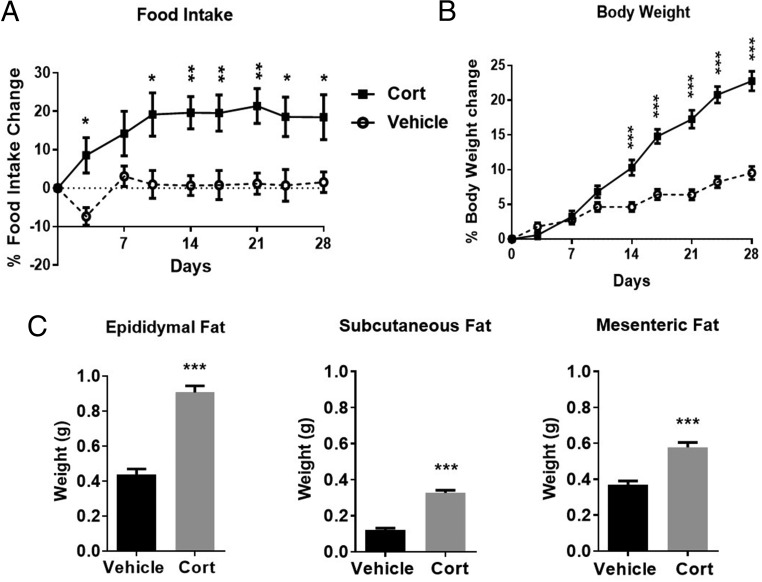
Exogenous Cort treatment induces hyperphagia and obesity. Food intake (A), body weight (B), and adiposity (C) after 4 weeks of exogenous Cort treatment; A and B, two-way ANOVA, and C, unpaired *t* test; ***, *P* < .001, n = 19/treatment group. Closed square represents Cort treated, open circle represents vehicle.

Associated with the rise in hypothalamic *Agrp* expression, food intake was increased after 3 days and remained elevated throughout the 4-week treatment (*P* < .01) (Figure 6A). Body weight significantly increased after 2 weeks of Cort compared with vehicle-treated animals (*P* < .001) (Figure 6B). Fat pad mass was also increased after 4 weeks of Cort treatment, with epididymal fat increased 2-fold, sc fat 2.6-fold, and mesenteric fat 1.5-fold (*P* < .001) (Figure 6C).

## Discussion

In this model of pharmacological treatment with Gcs to induce obesity, it is evident that Cort accumulates in the brain, and this study provides the first evidence that it is chronically elevated in the hypothalamus. This increase was seen after 24 hours and hypothalamic Cort levels remained elevated after 4 weeks of treatment, even though there was a reduction in Gc regeneration, suggesting a continuing direct influx of the administered Cort from the circulation. The increases in Cort were associated with elevated Gc activity in the hypothalamus, despite a decrease in hypothalamic GR. Treatment with Gcs increased the expression of the orexigenic neuropeptide, *Agrp*, in the hypothalamus and as might be expected there was increased food intake. At the end of the 4-week treatment period, mice had developed marked hyperphagia which could, in part, be driving the obesity.

We have used delivery of Cort in the drinking water, which represents a relatively stress-free approach of mimicking pharmacological administration of Gcs because of the implications of uncontrolled stress in the chronic paradigm. As mice eat and drink steadily throughout their active phase, in this model of Cort administration, they continue to take in a steady dose of Cort throughout the dark phase. A similar model was used by Karatsoreos et al, and although a higher dose of Cort (100 μg/mL) was given over 4 weeks, a comparable increase in circulating Cort was achieved (17). In the current study, the administration of Cort in the drinking water gave a greater increase in plasma Cort levels at ZT1 compared with ZT13. We believe this is due to mice starting their active phase at ZT12 and therefore eating and drinking at this time. As in this study, Karatsoreos et al (17) found that increased Cort caused adrenal atrophy during the treatment period, which is a marker of inhibition of HPA activity. However, they did not assess whether central effects of the elevated Cort might contribute to the body weight gain.

In the current study, treatment with Gcs resulted in markedly increased Cort in the hypothalamus, which remained chronically elevated throughout treatment. We cannot exclude the possibility that Cort from blood was present and quantified alongside tissue-specific Cort in both the hypothalamic and extrahypothalamic brain regions. However, studies have shown that blood contamination of whole-brain samples has little or no effect on brain steroid levels and saline-perfusions can be detrimental, because they can alter steroid concentrations in a region-specific manner (18). Although it is accepted that Gcs act in the hypothalamus to decrease corticotrophin-releasing hormone as part of the HPA axis, the concentrations of Gcs that exist after chronic Cort treatment have not previously been identified, in a model such as this. There are several mechanisms that exist to modify the levels and activity of Gcs. The efflux transporter (*Abcb1*) is able to remove Gcs from the brain (19), although it is unlikely to be effective in our model as its expression is not altered. There is also the 11β-HSD1 conversion of 11-DHC to Cort, which could be the main contributor to the levels of Cort in the hypothalamus and could be up-regulated as found in liver (20) and adipose tissue (3) or down-regulated to prevent further accumulation of Cort. In our study, there is decreased expression of hypothalamic *HSD11b1*, implying a protective mechanism, whereby with excess Gcs, there is a down-regulation of the enzyme to prevent regeneration of additional Cort from inactive 11-DHC. This indicates that increased hypothalamic Gc levels are more likely a result of the increased circulating Cort translocating into the brain and not a consequence of increased Cort regeneration. Further, the mechanisms controlling Cort entry and clearance may differ between brain regions as indicated by the lower Cort concentration in the hypothalamic region compared with extrahypothalamic region.

Perhaps more importantly, it is dogma from the HPA axis that chronic Gcs down-regulate the GR in the pituitary and hypothalamus. In the HPA axis, the reduction in GR is considered a compensatory mechanism to counteract the feedback inhibition by Gcs (21). We have found it hard to find any literature pertaining to this in a similar model to ours. Indeed, our novel findings show that Gcs do down-regulate the GR in our model, but most importantly, the receptor is not completely down-regulated. Gcs can still act in the hypothalamus to increase known target genes such as GILZ, a transcription factor, known to be sensitive to Gc activation (22, 23), and also maintain the decreases in 11β-HSD1 and GR. Although there is a 50% decrease in GR expression at 4 weeks, GILZ, is increased at 24 hours (our unpublished data) and remains elevated after 4 weeks of treatment. Therefore, the chronic exogenous treatment of Gcs down-regulates GR in our model, but most importantly, this partial down-regulation allows the continued action of Gcs in the hypothalamus to increase known target genes such as GILZ.

The increased hypothalamic Gcs, led us to investigate their effect on energy-regulating neuropeptides within the hypothalamus. Surprisingly, we did not see any change in *Pomc* mRNA in the hypothalamus by qRT-PCR or in situ hybridization. There are well-known mechanisms whereby Gcs inhibit pituitary-derived POMC. The binding of the GR to GREs in the promoter region of the POMC gene has been expertly mapped (24). However, the impact of Gc regulation on the neuron-specific promoter regions of POMC (25), which regulate hypothalamic POMC expression is less clear (4, 5, 26).

In a previous study, we observed an increase in *Agrp* expression in the hypothalamus with 25-μg/mL Cort in drinking water but the mice did not develop hyperphagia or obesity (7). In the current study with 75-μg/mL Cort, in drinking water, there was a clear increase in *Agrp* expression, and this was associated with the development of hyperphagia and obesity. It has previously been shown that fasting induces increases in Gcs, which activate transcription of the *Agrp* gene via GR binding directly to the AgRP-GRE (9). The elevated *Agrp* expression in our model, without alterations in other hypothalamic neuropeptide expression, suggests the importance of AgRP in central Gc signaling. Although AgRP neurons also release orexigenic γ-aminobutyric acid and NPY (27, 28), our studies suggest that Gcs do not increase NPY in this model. Given that NPY is predominantly expressed in the arcuate nucleus, it is unlikely that Gcs are modifying the NPY neurons that act on energy balance, but we cannot discount the fact that they may be causing changes in other regulatory functions in other hypothalamic regions.

In the current model, the effect of chronic treatment with Gcs is to increase hypothalamic Gcs, and compensatory mechanisms in Gc regulation do not lead to down-regulation of the system but rather allow a sustained enhanced effect of Gcs in the hypothalamus. This leads us to speculate that Gc-induced increases in *Agrp* may be mediating peripheral mechanisms alongside the development of hyperphagia leading to the development of obesity. Therefore, it is highly likely that Gc actions in the brain and periphery are not mutually exclusive. Indeed, although Gcs have well-recognized peripheral effects in regulating metabolism, this study suggests that chronically elevated Gcs acting in the hypothalamus may be the cause of abnormalities in neuronal regulation of energy balance that contribute to the plethora of metabolic side-effects.
